# Collateral damage and CRISPR genome editing

**DOI:** 10.1371/journal.pgen.1007994

**Published:** 2019-03-14

**Authors:** Mark Thomas, Gaetan Burgio, David J. Adams, Vivek Iyer

**Affiliations:** 1 Wellcome Sanger Institute, Wellcome Genome Campus, Hinxton, Cambridge, United Kingdom; 2 Department of Immunology and Infectious Disease, The John Curtin School of Medical Research, The Australian National University, Canberra, Australia; University of California Berkeley, UNITED STATES

## Abstract

The simplicity and the versatility of clustered regularly interspaced short palindromic repeats/CRISPR-associated protein (CRISPR-Cas) systems have enabled the genetic modification of virtually every organism and offer immense therapeutic potential for the treatment of human disease. Although these systems may function efficiently within eukaryotic cells, there remain concerns about the accuracy of Cas endonuclease effectors and their use for precise gene editing. Recently, two independent reports investigating the editing accuracy of the CRISPR-Cas9 system were published by separate groups at the Wellcome Sanger Institute; our study—Iyer and colleagues [1]—defined the landscape of off-target mutations, whereas the other by Kosicki and colleagues [2] detailed the existence of on-target, potentially deleterious deletions. Although both studies found evidence of large on-target CRISPR-induced deletions, they reached seemingly very different conclusions.

## So, what do scientists using CRISPR gene-editing technology need to know?

### Off targets—the need for controls

Iyer and colleagues used whole-genome sequencing (WGS) to identify potential off-target damage in mouse embryos and assessed the impact of genomic variation on de novo mutation calls. This study was undertaken in response to Schaefer and colleagues [3]—subsequently retracted—which reported a greater number of mutations at unexpected off-target locations in mice following the delivery of CRISPR reagents in mouse zygotes. Iyer and colleagues emphasised proper experimental controls and use of best practice in the choice of a single-guide RNA (sgRNA) and Cas9-mediated mutagenesis conditions. We demonstrated that if Cas9-mediated mutagenesis were causing off-target mutations, then the rate of these mutations was not distinguishable from the background de novo mutation rate in mouse embryos. The study concluded that efficient CRISPR gene editing was possible without a significant increase in de novo mutation rates, supporting the development of CRISPR-Cas9 as a therapeutic tool.

### On target—damage closer to home

Weeks later, Kosicki and colleagues reported unexpected on-target alterations at target locations in mouse embryonic stem cells, hematopoietic progenitors, and a differentiated human cell line. Although the vast majority of the CRISPR-induced double strand breaks (DSBs) resulted in small indels (<50 nt), up to 20% of editing events resulted in significantly larger deletions (>250 nt) and more complex genomic rearrangements than previously reported [4]. Using Pacific Biosciences (California) long-read sequencing and long-range PCR (over 5 kb), some of these events were shown to extend up to several kilobases from the protospacer adjacent motif (PAM) at the target site. The authors posited that these events would likely be missed using standard genotyping methods. Because this observation has significant implications for both research and therapeutic applications, the authors correctly concluded that comprehensive genomic analyses are warranted to fully characterise CRISPR-targeted cells.

### Are these studies directly comparable?

Not really. They focus on fundamentally different aspects of CRISPR-Cas9 genome editing, specifically on-target versus off-target damage, within different biological contexts. Iyer and colleagues characterized on-target and putative off-target alleles in mouse embryos, whereas Kosicki and colleagues only characterized on-target mutations in pooled cell assays.

### On-target mutation rates are comparable

With only 10 embryos, Iyer and colleagues lacks the statistical power to conclude anything more about large on-target deletions, other than that they occur with a frequency of at least 10%. Specifically, all 10 of the CRISPR-edited zygotes examined by Iyer and colleagues had an average of 2 mutant on-target alleles per embryo; 21 mutant alleles in total. Of these 21 alleles, 20 were “small” (<50 nt) deletions, detected with the bcftools small-variant caller. In one embryo, a large 338 nt deletion was also detected using Pindel, a structural variant caller. By contrast, Kosicki and colleagues only analyzed on-target effects. At the target *PigA* locus, the inferred overall proportion of “large” (>250 nt) alleles observed in the pool of cells from Kosicki and colleagues was approximately 20% across all sgRNAs, in which each distinct deletion is represented in a small number of cells.

### No data to compare for off-target mutation rates

Even though Kosicki and colleagues did not study off-target events, it could be inferred that large deletions or rearrangements at potential off-target locations may be missed by the WGS approaches used to analyze these events in Iyer and colleagues. The Pindel structural variant caller used to analyze all WGS reads from treated embryos in Iyer and colleagues identified one large de novo candidate off-target deletion of 260 nt. There was, however, no coincidence between this large deletion and any potential off-target site, and the deletion was therefore discounted as a potential off-target effect; we stand by this conclusion. Because the Cas9 ribonucleoprotein (RNPs) complexes are unlikely to persist beyond the two-cell stage, due to its short half-life, any CRISPR-induced mutations (including any large deletions) should be well represented in the mouse zygote. As such, the frequency of these mutations should have been clearly detected in the filtered variant calls from Iyer and colleagues, although none were observed.

### Zygote microinjection and pool transfection are different experiments

It is important to note that there are several technical and biological reasons why editing outcomes might be different between these two experimental approaches. The most notable difference is the synchronized and nonsynchronized cell cycle statuses of the targeted cells. Cytoplasmic microinjection was used by Iyer and colleagues to mutate fertilized single-cell mouse zygotes (confirmed by the presence of 2-pronuclei) with Cas9 RNP complexes. Although the exact cell cycle stage will have varied between each zygote, they are synchronized, because all microinjections were completed prior to coalescence of the pronuclei and therefore the completion of S-phase [5]. Any CRISPR-induced DSBs that occur at these early stages of the cell cycle are likely to be repaired by nonhomologous end joining (NHEJ), which is consistent with the formation of the small indels that were predominantly observed. By contrast, *PiggyBac* transposon-mediated delivery was predominantly used by Kosicki and colleagues to introduce constitutively active CRISPR reagents, although lipofection and electroporation methods were also used to deliver transient Cas9 RNPs into pools of mitotically active cells. Unlike Iyer and colleagues, however, the cells within these pools were not synchronized and DNA damage was likely to have occurred at different stages of the cell cycle, potentially altering DNA-repair outcomes. Indeed, cell cycle synchronization has previously been shown to improve RNP-mediated homology-directed repair (HDR) rates, although the effect on nontemplate DSB repair outcomes is less clear [6]. The importance of considering cell cycle changes was further demonstrated by Gu and colleagues, who exploited an extended G2-S phase at the two-cell stage in mouse zygotes to significantly increase the knock-in efficiency of large DNA fragments [7]. Furthermore, fundamental differences between the nuclear organization of single-cell embryos and mitotically active cells have also been noted by a recent study [8], which observed that there is a physical separation of the maternal and paternal genomes in early stage mouse zygote development. Both genomes exist on separate spindles, which are only aligned at the start of anaphase, prior to cleavage at the two-cell stage. The persistence of distinct maternal and paternal genomes until at least the two-cell stage may impair the formation of more complex rearrangements, including the apparent interhomologue repair (IHR) observed by Kosicki and colleagues at the *Cd9* locus in F1 C57BL/6 × CAST/Ei embryonic stem (ES) cells. Although the nuclear organization within single-cell zygotes may not favor IHR events, this does not entirely exclude the possibility of them occurring, as demonstrated by a recent study that used RAD51-enhanced IHR to increase the efficiency of homozygous knock-in insertions in mouse zygotes [9].

We have summarized the differences in on- and off-target data, along with experimental methodologies, in Table 1.

**Table 1:** Comparison of experimental methodologies and results.

Criteria	Iyer and colleagues	Kosicki and colleagues
Transfection method	Cytoplasmic microinjection	Piggybac, lipofection, electroporation
Cells mutated	Single-cell mouse zygotes, 2 pronuclei.	Mixed-phase pools of mouse ES cells or human RPE1 cells.
Cas9 + sgRNA delivery	Cas9 RNP	Cas9 RNP
Observed large on-target deletions?	Yes, at least 10% of zygotes (limited data).	Yes, at 20% of the cell population.
Observed large off-target deletions?	One large de novo deletion observed at non-target locus, unlikely to be true off-target.	No data.

**Abbreviation:** ES, embryonic stem.

### Supporting studies

Although the results of Iyer and colleagues are based on the targeting of a single gene, other similar studies have subsequently been published that support these findings. Using trios containing parents and embryos, these studies aimed to remove any variation within an animal colony, to achieve a better estimate of the de novo mutation burden in treated animals. The most recent study used a very similar approach to Iyer and colleagues—treating mouse zygotes with individual sgRNAs [10]. Using WGS of trios, they were able to compare de novo mutations in the CRISPR-treated mice to their untreated littermates. Like Iyer and colleagues, no appreciable increase in the de novo mutation burden of treated animals was observed when compared with untreated controls. In another study, WGS was used to analyse trios of Cas9-edited monkeys [11]. In this case, two separate experiments were performed, with potential off-target effects assessed in both by comparing the location of de novo mutations with predicted off-target locations, and by comparing the de novo mutation burden as a function of Cas9 editing efficiency, which was used as a surrogate for untreated control animals. As described for the above-mentioned mouse experiments, there was no association between Cas9 editing efficiency and de novo mutation burden. Moreover, as the monkey editing experiments generated both knock-out and knock-in alleles, the presence of an HDR template does not appear to have an effect on off-target activity. This does not, however, exclude the fact that off-target mutations can occur, as demonstrated by deep-sequencing analysis of 81 gene editing experiments in mouse and rat [12]. Detailed analysis of 10 mouse embryos and their genetic parents identified 43 true Cas9-generated off-target mutations consisting of small insertions or deletions. Although a considerable number of off-target mutations were detected, the authors acknowledge that this probably represents a worst-case scenario, because the specificity score of the sgRNA used in this experiment was very low. Collectively, these and other studies [13] highlight the importance of controlling for the effect of confounding genetic variation within a colony of animals when seeking to identify possible off-target mutations.

An elegant alternative approach to WGS is circularization for in vitro reporting of cleavage effects by sequencing (CIRCLE-seq) [14], an in vitro screening method that uses short-read sequencing of circularized sheared genomic DNA to identify CRISPR-induced DNA damage at all susceptible sites; both on- and off- target. As expected, the number of off-target mutations identified by CIRCLE-seq increases with the number of sgRNA mismatches with a promiscuous sgRNA generating multiple off-targets both in vitro and in vivo in somatically edited mice [15].

Regarding Kosicki and colleagues, it should be noted that large on-target deletions have also been observed at a low frequency in mice in some circumstances following treatment of single-cell mouse zygotes with an individual sgRNA [16, 17]. Unlike Iyer and colleagues, these studies microinjected Cas9 mRNA into the cytoplasm, which may have extended the activity of the Cas9 protein beyond the two- or four-cell stage. The potential outcomes of CRISPR-induced DSBs at these early stages in the developing zygote are therefore highly context- and method-dependent.

## Best-practice to reduce the risk of unwanted CRISPR damage

### sgRNA selection criteria for minimising unwanted on-target damage

As demonstrated in Kosicki and colleagues, the potential DNA-repair outcomes resulting from individual CRISPR-induced DSBs may be significantly larger and more complex than previously anticipated. Although the full extent of these outcomes remains to be determined, these events are likely to be highly context-dependent. In a recent study that investigated the targeting outcomes for more than 1,000 sgRNAs, nucleotides within the target sequence at the −2 to −5 position relative to the PAM were found to be critical for defining the editing precision [18]. The composition of the −4 nucleotide position adjacent to the cleavage site was particularly significant, with either an “A” or “T” frequently associated with a nucleotide insertion. Conversely, editing outcomes at target sites with a “G” nucleotide at the −4 position were found to be the most imprecise, inducing a variety of unpredictable deletions. Similar results were observed in a larger study, involving more than 40,000 sgRNAs, in which cell-line–specific differences were observed [19]. These studies have derived methods for predicting Cas9 editing outcomes based on nucleotide composition context at the target site. With the further development of such tools, it may be possible to bias DNA-repair outcomes in favor of smaller NHEJ-associated indels, as opposed to larger deletions associated with either microhomology-mediated end joining (MMEJ) or homologous recombination (HR).

### sgRNA selection criteria for minimizing off-target damage

Reducing the risk of on-target deletions does not alter the fact that complex rearrangements or mutations at DSBs might occur at off-target as well as on-target locations. Indeed, it is the innate ability of the native CRISPR-Cas systems to recognize both the target sequence and highly similar sequences [20] that raises the most concerns for CRISPR applications. As shown by Iyer and colleagues, it is possible to mitigate potential off-target effects by selecting sgRNAs with minimal potential off-target sites, as demonstrated by the specific targeting of the *Tyr* locus.

It may not always be possible to select sgRNAs with minimal off-targets, because a target region can impose specific constraints. Regions that are either repetitive or duplicated—such as with pseudogenes, paralogous gene expansions, or copy number variants—can result in sgRNAs with a much higher predicted off-target burden. These sgRNAs are likely to recognize highly similar sequences in other genomic regions that might contain as few as one or two nucleotide differences. Although such sgRNAs are unlikely to be used for therapeutic applications, they have been identified within pooled CRISPR libraries. These sgRNAs may confound the analysis of whole-genome screens, influencing results in a cell-line–specific manner that can lead to false positives and biased essentiality scores [21].

Finally, the current tools used for predicting off-targets are primarily based on the analysis of reference genome assemblies, which, although sufficient for most research purposes, will need to include personal genome variation for therapeutic applications. Such applications can be expected to employ highly annotated sgRNAs with well-defined off-target profiles that could potentially exclude highly sensitive genomic regions, such as tumor suppressor loci.

### Cas9 specificity

Although sgRNA selection is a critical component for minimizing off-target effects, it is also dependent on the specificity of the Cas9 endonuclease. Because the native Cas9 endonucleases are known to tolerate mismatches [22, 23], there have been significant efforts to engineer improved versions of the Cas9 endonuclease with increased on-target–binding specificity and reduced off-targets [24–27]. However, a consequence of this increased specificity is that the overall activity of the higher fidelity Cas9 versions can be diminished for some sgRNA targets [28]. This is sure to be an area in which further advances are made in the coming years.

### Genotyping

Of course, none of these practices will entirely exclude the possibility of unwanted damage occurring at either on-target or off-target locations, hence the requirement for in-vivo characterization of sgRNAs in either mouse zygotes or defined cell lines. This should include comprehensive genotyping with correct controls, either with WGS as described by Iyer and colleagues or CIRCLE-seq [14]. Although the larger structural variation highlighted by Kosicki and colleagues may be less common, multiple DSBs within the same chromosome associated with either on-target or combined on- and off-target activity can result in complex rearrangements [29]. In these instances, further validation using long-range genotyping approaches (for review, see [30]) will be required, as suggested by Kosicki and colleagues.

### Avoiding DSBs

Although all of these approaches are aimed at limiting the impact of unwanted effects associated with CRISPR-induced DSBs, it will ultimately be preferable to avoid, where possible, the in vivo cleavage of DNA in future therapeutic applications. Depending on the required outcome, it may be possible to alter the genome without creating DSBs, using either CRISPRa/i [31], epigenome-editing [32], base-editing [33], or RNA-editing [34] approaches. If DNA cleavage is required, an ex vivo approach may be used to edit a patient's cells under laboratory conditions. Under these circumstances, comprehensive genotyping should be used to confirm the absence of unintended mutations and off-target effects before these cells are returned to the patient.

### Take home message

Genome editing can be used with precision to engineer the genome, by following best practices. Quoting Professor Rodolphe Barrangou, “Keep calm and CRISPR on” [35].
